# *N*-*p*-Tolyl-1,3-selenazolo[5,4-*b*]pyridin-2-amine

**DOI:** 10.1107/S1600536813023659

**Published:** 2013-09-04

**Authors:** Zhaojun Wu, Yiqun Li, Hua Zhou, Meiyun Zhou

**Affiliations:** a Department of Chemistry, Jinan University, Guangzhou 510632, People’s Republic of China; b Department of Food Science and Technology, Jinan, University, Guangzhou 510632, People’s Republic of China

## Abstract

In the title compound, C_13_H_11_N_3_Se, the dihedral angle between the mean plane of the fused seleno­azolo­pyridine ring system and the *p*-toluidine ring is 14.260 (5)°. In the crystal, mol­ecules are linked by N—H⋯N hydrogen bonds, forming zigzag chains extending along the *b*-axis direction.

## Related literature

For the bioactivity of organoselenium, see: Garud *et al.* (2007 ▶); Ling *et al.* (2010 ▶); Plamen *et al.* (2010 ▶). For crystallographic studies on selenazolo derivatives, see: Plamen *et al.* (2004 ▶).
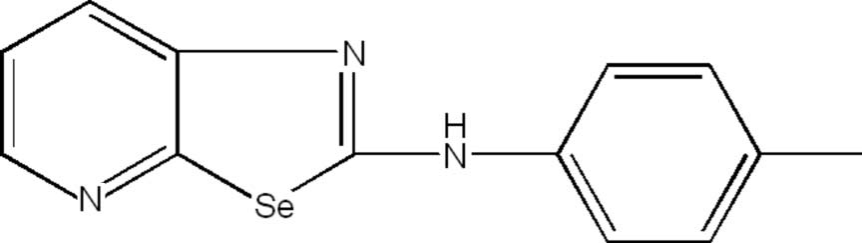


## Experimental

### 

#### Crystal data

C_13_H_11_N_3_Se
*M* *_r_* = 288.21
Orthorhombic, 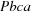

*a* = 13.138 (2) Å
*b* = 10.0323 (19) Å
*c* = 17.838 (3) Å
*V* = 2351.2 (7) Å^3^
*Z* = 8
Cu *K*α radiation
μ = 4.15 mm^−1^
*T* = 153 K
0.30 × 0.30 × 0.20 mm

#### Data collection

Agilent Xcalibur Sapphire3 Gemini ultra diffractometer
Absorption correction: multi-scan (*CrysAlis PRO*; Agilent, 2010 ▶) *T*_min_ = 0.369, *T*_max_ = 0.491
5248 measured reflections
1863 independent reflections
1423 reflections with *I* > 2σ*I*)
*R*_int_ = 0.064

#### Refinement

*R*[*F* ^2^ > 2σ(*F* ^2^)] = 0.094
*wR*(*F* ^2^) = 0.314
*S* = 1.28
1863 reflections
155 parameters
H-atom parameters constrained
Δρ_max_ = 1.47 e Å^−3^
Δρ_min_ = −2.49 e Å^−3^

### 

Data collection: *CrysAlis PRO* (Agilent, 2010 ▶); cell refinement: *CrysAlis PRO*; data reduction: *CrysAlis PRO*; program(s) used to solve structure: *SHELXS97* (Sheldrick, 2008 ▶); program(s) used to refine structure: *SHELXL97* (Sheldrick, 2008 ▶); molecular graphics: *SHELXTL* (Sheldrick, 2008 ▶); software used to prepare material for publication: *publCIF* (Westrip, 2010 ▶) and *PLATON* (Spek, 2009 ▶).

## Supplementary Material

Crystal structure: contains datablock(s) I, New_Global_Publ_Block. DOI: 10.1107/S1600536813023659/zs2275sup1.cif

Structure factors: contains datablock(s) I. DOI: 10.1107/S1600536813023659/zs2275Isup2.hkl

Supplementary material file. DOI: 10.1107/S1600536813023659/zs2275Isup3.cml

Additional supplementary materials:  crystallographic information; 3D view; checkCIF report

## Figures and Tables

**Table 1:** Hydrogen-bond geometry (Å, °)

*D*—H⋯*A*	*D*—H	H⋯*A*	*D*⋯*A*	*D*—H⋯*A*
N10—H10⋯N1^i^	0.88	2.11	2.983 (16)	175

Symmetry code: (i) 
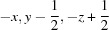
.

**Fig. 1.**
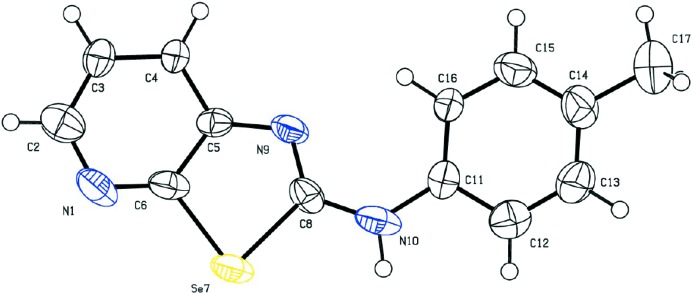
The molecular structure of the title compound showing the atom numbering scheme. Displacement ellipsoids are drawn at the 50% probability level.

## supplementary crystallographic information

### 1. Comment

Since the discovery of the importance of Se as a microelement in bacteria and animals, and the function of the selenoenzyme glutathione peroxidase (GPx) as an antioxidant, the interest in organoselenium compounds has increased significantly (Garud *et al.*, 2007; Ling *et al.*, 2010; Plamen *et al.*, 2004, 2010). The design and synthesis of organoselenium compounds, especially Se-containing heterocycles, are our current interest. The title molecule, C_13_H_11_N_3_Se, (Fig. 1) is built up from two fused rings, *viz*. the selenazolo and pyridine rings, linked to a *p*-toluidine group. The dihedral angle between these two ring systems is 14.260 (5)°. In the crystal, the molecules are linked by intermolecular N—H···N hydrogen bonds (Table 1), giving one-dimensional zigzag chains extending along *b*.

### 2. Experimental

To a stirred solution of *N*-*p*-tolylformamide (10 mmol) in toluene (100 ml) in an ice bath, Et_3_N (4.0 g, 40 mmol) and Se black powder were added. Phosgene (8 g of a 20% solution in toluene) was then added slowly over 30 min. giving an exothermic reaction. After complete addition, the suspension was heated under reflux for 10 h (TLC control). The mixture was filtered and washed with several portions of toluene, and the filtrate was then concentrated, affording the raw isoselenocyanatobenzene. This was added to a stirred solution of 2-chloropyridin-3-amine (1.28 g, 10 mmol) in 2-propanol at room temperature, and the mixture was heated to reflux for 6 h. After filtration, the precipitate was collected as a yellow solid. The impure product was dissolved in CCl_2_H_2_ at room temperature. Yellow crystals suitable for X-ray analysis (80.2% yield) grew over a period of one week when the solution was exposed to the air.

### 3. Refinement

Hydrogen atoms were placed at calculated positions [N—H = 0.88 Å, C—H(aromatic) = 0.95 Å, C—H(methyl) = 0.98 Å] and treated as riding, with *U*_iso_(H) = 1.2*U*_eq_(N and aromatic C) and *U*_iso_(H) = 1.5*U*_eq_(methyl C). A check using *TwinRotMat* within *PLATON* (Spek, 2009) detected no twin law.

### Crystal data

**Table:**

C_13_H_11_N_3_Se	*F*(000) = 1152
*M**_r_* = 288.21	*D*_x_ = 1.628 Mg m^−^^3^
Orthorhombic, *P**b**c**a*	Cu *K*α radiation, λ = 1.54178 Å
Hall symbol: -P 2ac 2ab	Cell parameters from 7867 reflections
*a* = 13.138 (2) Å	θ = 1.3–61.9°
*b* = 10.0323 (19) Å	µ = 4.15 mm^−^^1^
*c* = 17.838 (3) Å	*T* = 153 K
*V* = 2351.2 (7) Å^3^	Prism, yellow
*Z* = 8	0.30 × 0.30 × 0.20 mm

### Data collection

**Table:**

Agilent Xcalibur Sapphire3 Gemini ultra diffractometer	1863 independent reflections
Radiation source: fine-focus sealed tube	1423 reflections with *I* > 2s˘*I*)
Graphite monochromator	*R*_int_ = 0.064
Detector resolution: 16.0288 pixels mm^-1^	θ_max_ = 63.6°, θ_min_ = 6.0°
ω scans	*h* = −15→13
Absorption correction: multi-scan (*CrysAlis PRO*; Agilent, 2010)	*k* = −11→11
*T*_min_ = 0.369, *T*_max_ = 0.491	*l* = −13→20
5248 measured reflections	

### Refinement

**Table:**

Refinement on *F*^2^	Primary atom site location: structure-invariant direct methods
Least-squares matrix: full	Secondary atom site location: difference Fourier map
*R*[*F*^2^ > 2σ(*F*^2^)] = 0.094	Hydrogen site location: inferred from neighbouring sites
*wR*(*F*^2^) = 0.314	H-atom parameters constrained
*S* = 1.28	*w* = 1/[σ^2^(*F*_o_^2^) + (0.0796*P*)^2^ + 53.5115*P*] where *P* = (*F*_o_^2^ + 2*F*_c_^2^)/3
1863 reflections	(Δ/σ)_max_ < 0.001
155 parameters	Δρ_max_ = 1.47 e Å^−^^3^
0 restraints	Δρ_min_ = −2.49 e Å^−^^3^

### Special details

**Table:**

Geometry. All e.s.d.'s (except the e.s.d. in the dihedral angle between two l.s. planes) are estimated using the full covariance matrix. The cell e.s.d.'s are taken into account individually in the estimation of e.s.d.'s in distances, angles and torsion angles; correlations between e.s.d.'s in cell parameters are only used when they are defined by crystal symmetry. An approximate (isotropic) treatment of cell e.s.d.'s is used for estimating e.s.d.'s involving l.s. planes.
Refinement. Refinement of *F*^2^ against ALL reflections. The weighted *R*-factor *wR* and goodness of fit *S* are based on *F*^2^, conventional *R*-factors *R* are based on *F*, with *F* set to zero for negative *F*^2^. The threshold expression of *F*^2^ > σ(*F*^2^) is used only for calculating *R*-factors(gt) *etc*. and is not relevant to the choice of reflections for refinement. *R*-factors based on *F*^2^ are statistically about twice as large as those based on *F*, and *R*- factors based on ALL data will be even larger.

### Fractional atomic coordinates and isotropic or equivalent isotropic displacement parameters (Å^2^)

**Table:**

	*x*	*y*	*z*	*U*_iso_*/*U*_eq_	
C2	0.1440 (11)	0.5123 (17)	0.1134 (9)	0.058 (4)	
H2	0.1245	0.5846	0.0821	0.069*	
C3	0.2452 (11)	0.4817 (14)	0.1192 (8)	0.049 (3)	
H3	0.2937	0.5321	0.0917	0.058*	
C4	0.2779 (10)	0.3779 (13)	0.1647 (7)	0.042 (3)	
H4	0.3483	0.3586	0.1694	0.050*	
C6	0.1034 (9)	0.3460 (16)	0.1916 (7)	0.045 (3)	
C5	0.2051 (9)	0.3016 (13)	0.2038 (7)	0.035 (3)	
C8	0.1441 (10)	0.1537 (15)	0.2778 (7)	0.041 (3)	
C11	0.2188 (10)	−0.0164 (13)	0.3649 (7)	0.038 (3)	
C16	0.3195 (10)	0.0139 (14)	0.3591 (8)	0.045 (3)	
H16	0.3399	0.0836	0.3263	0.054*	
C15	0.3925 (11)	−0.0535 (18)	0.3993 (8)	0.059 (4)	
H15	0.4618	−0.0279	0.3942	0.071*	
C14	0.3684 (12)	−0.1555 (19)	0.4460 (8)	0.058 (4)	
C13	0.2672 (13)	−0.189 (2)	0.4510 (8)	0.064 (5)	
H13	0.2482	−0.2601	0.4834	0.077*	
C12	0.1923 (12)	−0.1245 (16)	0.4116 (7)	0.052 (4)	
H12	0.1234	−0.1523	0.4156	0.063*	
C17	0.4499 (16)	−0.2322 (19)	0.4884 (9)	0.074 (5)	
H17A	0.4471	−0.3265	0.4742	0.110*	
H17B	0.4380	−0.2236	0.5425	0.110*	
H17C	0.5170	−0.1960	0.4760	0.110*	
N1	0.0717 (9)	0.4456 (15)	0.1496 (7)	0.058 (4)	
N9	0.2255 (8)	0.1982 (14)	0.2518 (6)	0.049 (3)	
N10	0.1388 (8)	0.0493 (14)	0.3276 (7)	0.054 (3)	
H10	0.0772	0.0197	0.3375	0.065*	
Se7	0.01740 (10)	0.22670 (19)	0.24564 (9)	0.0504 (7)	

### Atomic displacement parameters (Å^2^)

**Table:**

	*U*^11^	*U*^22^	*U*^33^	*U*^12^	*U*^13^	*U*^23^
C2	0.048 (8)	0.060 (10)	0.066 (10)	0.007 (8)	−0.001 (7)	−0.012 (8)
C3	0.053 (8)	0.028 (7)	0.065 (9)	0.000 (6)	0.001 (7)	0.010 (7)
C4	0.038 (7)	0.035 (8)	0.052 (8)	0.001 (6)	0.000 (6)	0.015 (6)
C6	0.028 (6)	0.066 (10)	0.041 (7)	−0.006 (7)	−0.001 (5)	−0.006 (7)
C5	0.031 (6)	0.032 (7)	0.043 (7)	−0.006 (5)	0.001 (5)	−0.007 (6)
C8	0.036 (7)	0.048 (8)	0.040 (7)	0.016 (6)	0.002 (6)	−0.005 (6)
C11	0.051 (8)	0.032 (7)	0.031 (6)	0.006 (6)	0.005 (5)	−0.003 (6)
C16	0.040 (7)	0.042 (8)	0.052 (8)	0.004 (6)	0.005 (6)	0.017 (7)
C15	0.039 (7)	0.082 (13)	0.056 (9)	0.003 (8)	0.004 (7)	0.004 (9)
C14	0.060 (9)	0.078 (12)	0.036 (7)	0.013 (8)	0.001 (6)	0.007 (8)
C13	0.067 (11)	0.084 (14)	0.041 (8)	−0.006 (9)	0.005 (7)	0.017 (9)
C12	0.055 (9)	0.059 (10)	0.043 (7)	−0.005 (8)	0.004 (7)	−0.006 (7)
C17	0.094 (14)	0.066 (12)	0.061 (10)	0.011 (10)	−0.010 (9)	0.007 (9)
N1	0.036 (6)	0.089 (11)	0.048 (7)	0.018 (7)	−0.007 (5)	0.000 (7)
N9	0.024 (6)	0.082 (10)	0.042 (6)	0.005 (5)	0.002 (4)	0.012 (6)
N10	0.025 (5)	0.082 (10)	0.054 (7)	−0.001 (6)	0.001 (5)	−0.009 (7)
Se7	0.0237 (9)	0.0734 (13)	0.0542 (10)	−0.0005 (7)	−0.0003 (6)	−0.0021 (8)

### Geometric parameters (Å, º)

**Table:**

C2—N1	1.33 (2)	C11—N10	1.408 (17)
C2—C3	1.37 (2)	C11—C12	1.41 (2)
C2—H2	0.9500	C16—C15	1.38 (2)
C3—C4	1.388 (18)	C16—H16	0.9500
C3—H3	0.9500	C15—C14	1.36 (2)
C4—C5	1.410 (17)	C15—H15	0.9500
C4—H4	0.9500	C14—C13	1.37 (2)
C6—N1	1.316 (19)	C14—C17	1.52 (2)
C6—C5	1.425 (18)	C13—C12	1.37 (2)
C6—Se7	1.907 (14)	C13—H13	0.9500
C5—N9	1.371 (17)	C12—H12	0.9500
C8—N9	1.248 (17)	C17—H17A	0.9800
C8—N10	1.375 (18)	C17—H17B	0.9800
C8—Se7	1.907 (12)	C17—H17C	0.9800
C11—C16	1.362 (19)	N10—H10	0.8800
			
N1—C2—C3	123.0 (16)	C14—C15—C16	121.9 (14)
N1—C2—H2	118.5	C14—C15—H15	119.1
C3—C2—H2	118.5	C16—C15—H15	119.1
C2—C3—C4	120.8 (14)	C15—C14—C13	116.8 (15)
C2—C3—H3	119.6	C15—C14—C17	121.6 (15)
C4—C3—H3	119.6	C13—C14—C17	121.6 (16)
C3—C4—C5	119.2 (12)	C12—C13—C14	123.0 (16)
C3—C4—H4	120.4	C12—C13—H13	118.5
C5—C4—H4	120.4	C14—C13—H13	118.5
N1—C6—C5	128.4 (12)	C13—C12—C11	119.3 (14)
N1—C6—Se7	125.2 (10)	C13—C12—H12	120.4
C5—C6—Se7	106.4 (10)	C11—C12—H12	120.4
N9—C5—C4	126.0 (11)	C14—C17—H17A	109.5
N9—C5—C6	121.0 (11)	C14—C17—H17B	109.5
C4—C5—C6	113.0 (12)	H17A—C17—H17B	109.5
N9—C8—N10	123.7 (12)	C14—C17—H17C	109.5
N9—C8—Se7	119.9 (11)	H17A—C17—H17C	109.5
N10—C8—Se7	116.3 (9)	H17B—C17—H17C	109.5
C16—C11—N10	125.7 (12)	C6—N1—C2	115.7 (12)
C16—C11—C12	117.2 (13)	C8—N9—C5	109.6 (11)
N10—C11—C12	117.1 (12)	C8—N10—C11	128.7 (11)
C11—C16—C15	121.8 (13)	C8—N10—H10	115.7
C11—C16—H16	119.1	C11—N10—H10	115.7
C15—C16—H16	119.1	C8—Se7—C6	82.9 (6)
			
N1—C2—C3—C4	−1 (2)	N10—C11—C12—C13	−177.9 (13)
C2—C3—C4—C5	2 (2)	C5—C6—N1—C2	1 (2)
C3—C4—C5—N9	−178.7 (13)	Se7—C6—N1—C2	−176.5 (11)
C3—C4—C5—C6	−1.3 (18)	C3—C2—N1—C6	−1 (2)
N1—C6—C5—N9	177.7 (14)	N10—C8—N9—C5	179.9 (12)
Se7—C6—C5—N9	−4.7 (15)	Se7—C8—N9—C5	2.3 (17)
N1—C6—C5—C4	0 (2)	C4—C5—N9—C8	179.0 (13)
Se7—C6—C5—C4	177.8 (9)	C6—C5—N9—C8	1.8 (18)
N10—C11—C16—C15	178.2 (14)	N9—C8—N10—C11	8 (2)
C12—C11—C16—C15	−3 (2)	Se7—C8—N10—C11	−174.5 (11)
C11—C16—C15—C14	1 (2)	C16—C11—N10—C8	1 (2)
C16—C15—C14—C13	0 (2)	C12—C11—N10—C8	−177.8 (13)
C16—C15—C14—C17	178.1 (15)	N9—C8—Se7—C6	−4.1 (12)
C15—C14—C13—C12	0 (3)	N10—C8—Se7—C6	178.1 (11)
C17—C14—C13—C12	−177.8 (15)	N1—C6—Se7—C8	−178.1 (13)
C14—C13—C12—C11	−2 (3)	C5—C6—Se7—C8	4.2 (9)
C16—C11—C12—C13	3 (2)		

### Hydrogen-bond geometry (Å, º)

**Table:**

*D*—H···*A*	*D*—H	H···*A*	*D*···*A*	*D*—H···*A*
N10—H10···N1^i^	0.88	2.11	2.983 (16)	175

Symmetry code: (i) −*x*, *y*−1/2, −*z*+1/2.
